# Modeling‐Facilitated Field Survey Discovers of a New Population of the Annamite Striped Rabbit in Kon Tum Province, Vietnam

**DOI:** 10.1002/ece3.70752

**Published:** 2024-12-23

**Authors:** Anh Tuan Nguyen, Minh Le

**Affiliations:** ^1^ Faculty of Environmental Sciences University of Science, Vietnam National University Hanoi Vietnam; ^2^ Central Institute for Natural Resources and Environmental Studies Vietnam National University Hanoi Vietnam

**Keywords:** camera trap, Maxent, *Nesolagus timminsi*, southern Annamite landscape, Vietnam

## Abstract

The Annamite striped rabbit ( *Nesolagus timminsi* ) is an Endangered lagomorph endemic to the Annamite Mountain Range in Vietnam and Laos, with much unknown about its distribution range. In this study, we used previously known records of the Annamite striped rabbit to construct distribution models using Maxent, a modeling approach that has been proven to be robust in identifying potential areas of undiscovered populations of targeted species. Using the optimal model results as a guideline to select the field site, we conducted a systematic camera trap survey in Dak Long Protection Forest, Kon Tum Province, Vietnam. We recorded the species in five events at two locations of the study site, and to the best of our knowledge this finding represents a new population of the rabbit in this region. Our discovery significantly expands the known range of the striped rabbit, and we discuss the implications of this finding for *N. timminsi* conservation. We also suggest several protected areas in the South of our discovery site that, according to our optimal model results, may harbor unknown populations of the Annamite striped rabbit, and hence they should be prioritized for future field surveys. Our finding also highlights the importance of modeling tools in biodiversity surveys, especially for elusive and poorly studied species.

## Graphical abstract

We present our discovery of a previously unknown population of the Annamite striped rabbit, an endangered lagomorph, in Central Highlands, Vietnam using results from species distribution model and camera trap survey. We recorded the species in five events at two locations of the study site, and to the best of our knowledge this finding represents a new population of the rabbit in this region.

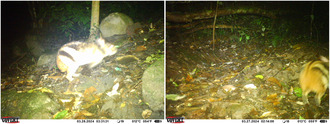

## 1 Introduction

The Annamite striped rabbit ( *Nesolagus timminsi* ) is an endemic to the region along the Annamite ecoregion lagomorph endemic to the region along the Annamite ecoregion between Vietnam and Laos (Tilker et al. 2020). The species was only known to science in the 1990s (Surridge et al. 1999), and for the next two decades all available information suggests that the small mammal is confined to the wet evergreen forest with limited prolonged dry season (Can et al. 2001; Duckworth, Salter, and Khounboline 1999; Nguyen, Wilting et al. 2022; Tilker et al. 2020). Before the 2020s, the species was recorded as far north as Nghe An Province in north‐central Vietnam and adjacent Bolikhamxay Province in Laos, and the southernmost known records were reported from Quang Nam Province in central Vietnam and neighboring Sekong Province in Laos (Abramov, Tikhonov, and Orlov 2016; Averianov, Abramov, and Tikhonov 2000; Can et al. 2001; Mahood and Hung van 2008).

Despite a number of studies on its ecology, distribution, and genetic diversity (Can et al. 2001; Tilker et al. 2020; Nguyen, Wilting et al. 2022), crucial knowledge gaps about the species persist. For instance, the actual distribution range of the species remains uncertain, as evidenced by the recent discoveries of previously unknown populations in northern Kon Tum and Lam Dong provinces, both of which are located in the Central Highlands of Vietnam (Nguyen et al. 2021, 2023). The new findings significantly expand the southern range of the rabbit. However, the current known distribution of *N. timminsi* in the Central Highlands seems to be highly fragmented, as other intensive camera trapping surveys in the region did not record the species in between the isolated locations (Tam et al. 2021; Wearn et al. 2021). Collectively, this dearth of information impedes the formulation of effective range‐wide conservation strategies for the endangered rabbit.

Species distribution model is an approach that has been used for a wide range of studies, including determining the potential geographical distribution of threatened species (Jafari, Zamani‐Ahmadmahmoodi, and Mirzaei 2018; Thapa et al. 2018; Yi et al. 2016), identifying stable refugia that may harbor rare species (Li et al. 2016; Tang et al. 2018), directing field surveys to discover new populations of endangered and endemic species (Udyawer et al. 2020; van Schingen et al. 2016), and studying speciation and evolutionary processes of sister taxa over a long timescale (Bett, Blair, and Sterling 2012). Maximum Entropy (Maxent) is a species distribution model approach that has been used extensively (Urbina‐Cardona et al. 2019), as it only requires the presence records for model construction (Elith et al. 2006; Phillips, Anderson, and Schapire 2006), and it also produces robust results even for species with a small number of known records (Pearson et al. 2007).

In this study, in 2023, we collated all available records of the Annamite striped rabbit (described in Section 2) and used the dataset to construct optimal Maxent models. Using information from Maxent results, in early 2024, we conducted a systematic camera trap survey in Dak Long Protection Forest, an under‐surveyed site, which lies about 30 km south of the recently discovered localities of the species in Ngoc Linh region (Nguyen et al. 2023).

## 2 Materials and Methods

### 2.1 Species Distribution Model

**FIGURE 1:**
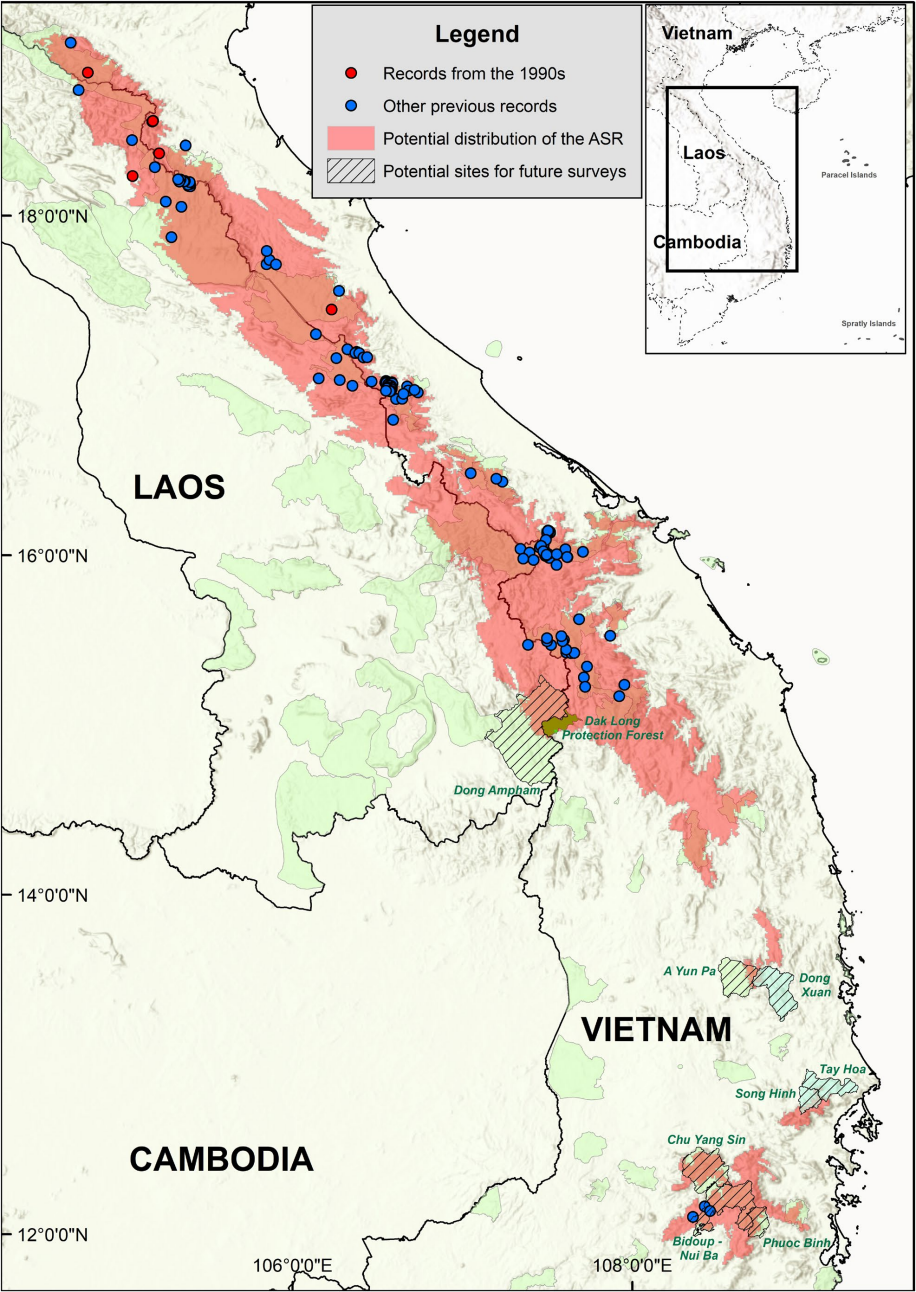
Maxent distribution model result for the Annamite striped rabbit in current conditions.

We reviewed available records of the Annamite striped rabbit by searching the Web of Science, Google Scholar, ResearchGate, GBIF, and iNaturalist using the following queries: “*Nesolagus*,” “ *Nesolagus timminsi* ,” “Annamite striped rabbit,” “striped rabbit,” and their local language equivalents such as “Thỏ vằn,” “Thỏ vằn Trường Sơn,” “Thỏ vằn Trung Bộ,” “ກະຕ່າຍລາຍຂີດອັນນາມິດ” and so on. In addition, library archives, reports, field notes, documents, and specimens from related museums and institutions were also examined. For localities that had no coordinates, and only had geographic feature information, we used protocol specified by Zermoglio et al. (2020) to determine the estimated localities. Localities from online platforms such as GBIF and iNaturalist were only used to assess the reliability of records from published papers and field reports, and were not used directly in the modeling process. In total, we collated 129 localities for the Annamite striped rabbit from both previous studies and from our previous field surveys (Figure 1, Table S1). The records were then checked, assessed, and filtered to avoid erroneous or inaccurate localities as recommended by Chapman (2005). To reduce spatial‐autocorrelation and possible sampling biases, we used the spThin package (Aiello‐Lammens et al. 2015) in (R Core Team 2023) to thin out localities within 10 km distance following recommendations from Pearson et al. (2007), reducing our dataset to a final set of 47 localities.

We constructed the species distribution models using all 19 bioclimatic variables at 30‐arcsecond resolution available at WorldClim 2.1 database (Fick and Hijmans 2017). Based on previous records (Nguyen et al. 2021), and to only sample the areas that are likely suitable for the species, we restricted the model extent to a 3.0° buffer around the occurrences (Anderson and Raza 2010). We used ENMTools (Warren and Seifert 2011) to calculate Pearson's correlation coefficient to identify highly correlated variables (*r* ≥ |0.60|) and a jackknife analysis to measure the importance of variables. After removing highly correlated variables, the final set of variables included mean diurnal range, temperature seasonality, mean temperature of warmest quarter, precipitation seasonality, precipitation of wettest quarter, precipitation of driest quarter, and precipitation of warmest quarter.

We ran all analyses in Maxent version 3.4.4 (Phillips et al. 2017). As Maxent tends to produce overfitting models, which may reduce its ability to predict possible new distribution regions (Merow, Smith, and Silander 2013), we performed the following steps of additional tuning decisions to minimize the overfitting issue and also maximize discriminatory ability. We performed the tuning process using the ENMeval R package (Kass et al. 2021) with all feature class combinations and tested a range of regularization multiplier values ranging from 0.5 to 10.0 by increments of 0.5. Other model parameters, for example, convergence threshold and background samples, followed recommendations from the model developers (Phillips, Anderson, and Schapire 2006). We then used the spatial fourfold cross‐validation method, which is recommended for models that need to be projected on novel conditions, to train the models (Kass et al. 2021).

To assess model performance, we used the 10% omission rate threshold to select models that showed the least overfitting and then chose models with the highest area under the receiver operating characteristic curve (AUC) values. From this subset, models were then compared using the Akaike information criterion (AIC), which balances complexity with model fitness (Warren and Seifert 2011). For the optimal model, we used the 10% training presence threshold to classify between suitable and unsuitable areas for the Annamite striped rabbit (Pearson et al. 2007).

### 2.2 Camera Trap Survey

**FIGURE 2:**
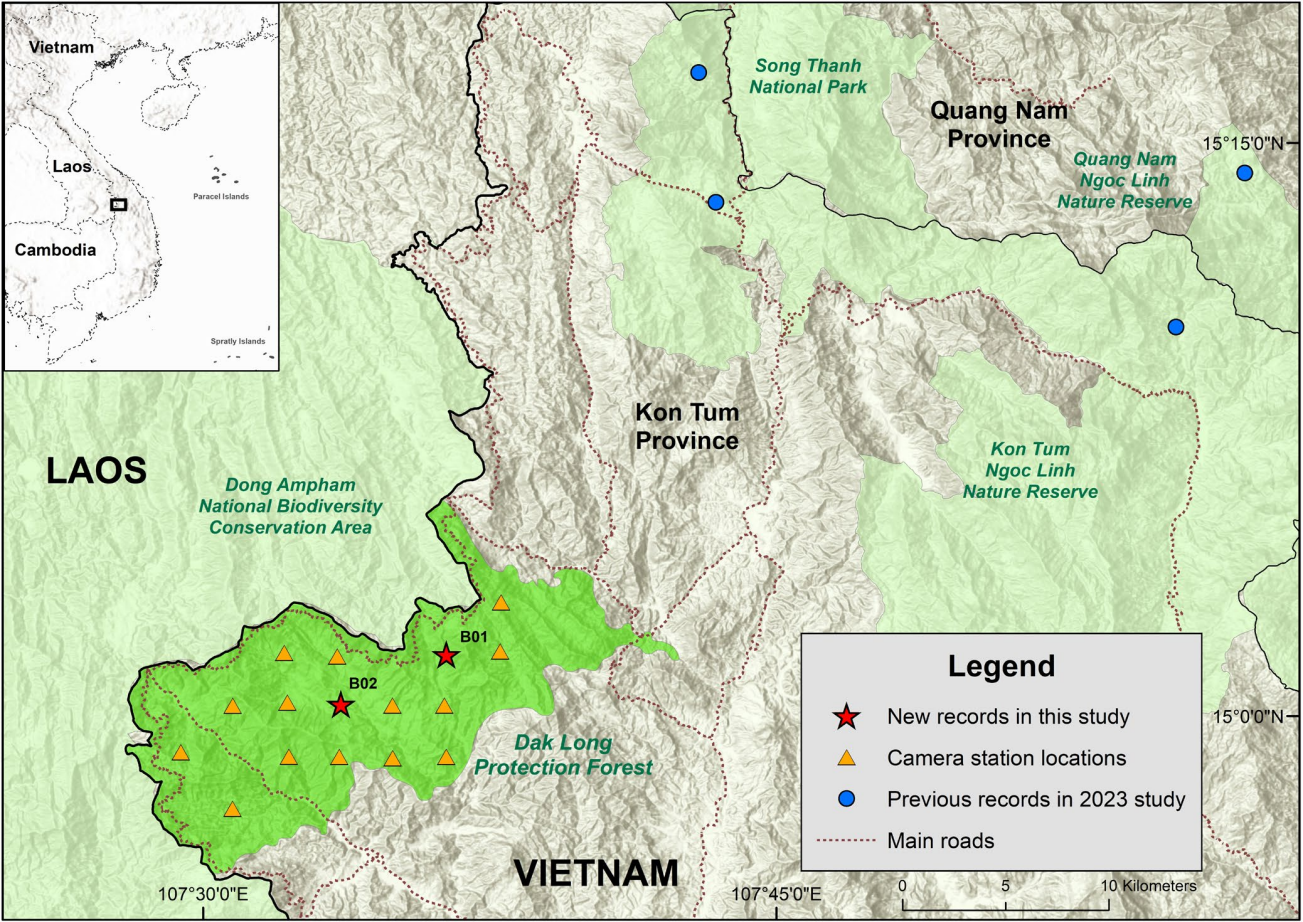
New records of the Annamite striped rabbit at the study site. The blue dots represent the recently discovered populations of the *N. timminsi* in Ngoc Linh area (Nguyen et al. 2023).

Based on the resulting optimal model, we screened a number of protected areas and protection forests in Vietnam using three criteria: (i) They had not had any previously confirmed records of the Annamite striped rabbit (Figure 1). (ii) They were situated within a large continuous suitable region according to the Maxent optimal model (Figure 1). (iii) They had not had any extensive camera trap surveys for the last 10 years (Nguyen et al. 2021; Tam et al. 2021; Wearn et al. 2021). Dak Long Protection Forest, covering approximately 15,000 ha from Kon Tum Province, the Central Highlands, Vietnam, was selected because it met all of the criteria. Dak Long Protection Forest has elevations from about 700 m to more than 1840 m, and there are multiple road systems than run around or even into the forests (Figure 2). The majority of the site was a mix of mature secondary forest, highly degraded scrub, and plantation forests. Isolated pockets of wet evergreen forest scattered across the region. There were a few human settlements and agriculture lands inside the protection forest, and signs of human disturbances, such as logged trees and camps, could still be found.

From 17 January 2024 to 23 April 2024, we conducted a systematic camera‐trap survey in the study site, with overall protocols that followed recommendations from Abrams et al. (2018). In particular, camera stations were spaced approximately 2.5 km apart in a systematic grid, and the minimum distance between any camera station pair was 2.0 km. Two independent white‐flash camera traps (Covert Illuminator) were set at each station, with cameras facing different directions, and were positioned along wildlife trails to maximize animal detections (Abrams et al. 2018). Cameras were set 20–40 cm above the ground and programmed to take a three‐photo burst when triggered. A total of 16 camera stations were set up across the survey area, but cameras were stolen or destroyed at two stations, and thus we were only able to retrieve 14 stations. We used camtrapR (Niedballa et al. 2016) to process the data. When processing the data, we treated the two cameras at the same location as a single station, and a 60‐min interval was employed to define independent detections at any given station (Figure 2).

## 3 Results

### 3.1 Species Distribution Model

The Maxent's optimal model had a regularization multiplier value of 2.5 and a linear and quadratic combination feature class. The model demonstrated good predictive performance for the Annamite striped rabbit's distribution, with an average value for training AUC of 0.936, validation AUC of 0.924, and omission rate of 0.089. All final models predicted highly similar overall distributions of *N. timminsi* , and they only had minor differences in exact location details and the area of suitable regions. Spatially, the optimal model encompassed the known localities of the Annamite striped rabbit (Figure 1).

Maxent also provided an assessment of the most important variables used in the model through variable contribution. The variables with highest contribution were BIO17 (Precipitation of driest quarter), BIO10 (Mean temperature of warmest quarter), BIO18 (Precipitation of warmest quarter), and BIO02 (Mean diurnal range).

### 3.2 Camera Trap Results

The cameras were deployed for an average of 92 ± 4.6 days. We recorded the Annamite striped rabbit at two stations, both of which were located only a few kilometers from Vietnam—Laos border (Figure 2). In addition to the *N. timminsi* , we also documented other mammals at the stations, including the Asiatic brush‐tailed porcupine ( *Atherurus macrourus* ), common palm civet ( *Paradoxurus hermaphroditus* ), crab‐eating mongoose (*Urva urva*), stump‐tailed macaque ( *Macaca arctoides* ), ferret‐badger (*Melogale* spp.), Pallas' squirrel ( *Callosciurus erythraeus* ), red‐cheeked squirrel ( *Dremomys rufigenis* ), and yellow‐throated marten ( *Martes flavigula* ). The most common soil type in the site is feralite soil on magmatic rock, and the site is generally characterized by a mix of both wet evergreen forests, as well as mature and regenerating secondary forests. The distance from the camera stations to the closest human settlements is just around seven kilometers. While the terrain between two stations and nearby villages are highly dissected with multiple ridgelines, there is a road than runs through Dak Long, and the distances from both stations to that road are significantly shorter, ranging from 1.5 to 2.5 km (Figure 2).

**FIGURE 3:**
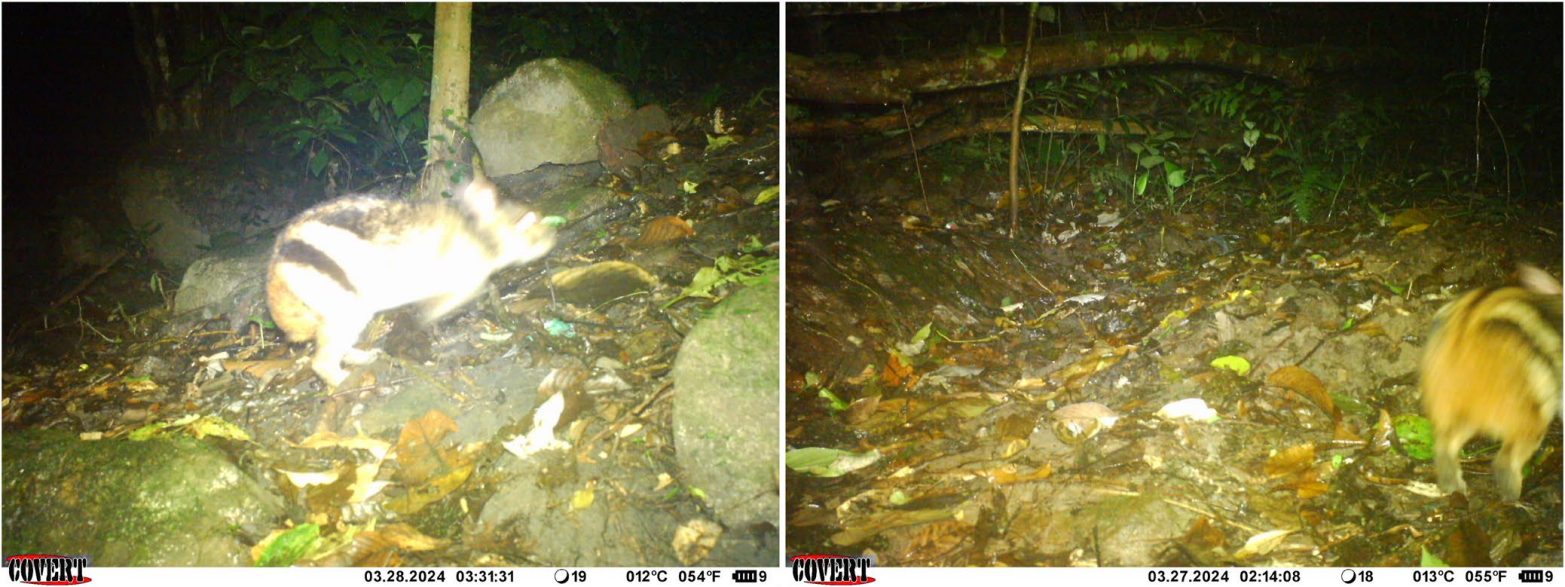
Annamite striped rabbits recorded during our survey.

In total, the Annamite striped rabbit was detected at five independent events (Figure 3). Both recorded stations were located in a relatively flat area with a nearby running stream, and the surrounding habitat type was mostly wet evergreen forest with dense, moist understory vegetation. They were set up just a few kilometers from a road that runs along the Vietnam—Laos border and dissects through the protection forest (Figure 2). In each station, only one camera recorded the species. The first camera station (B01) was active for 94 days and only recorded *N. timminsi* once. The second camera station (B02) was active for 91 days and captured four independent events of the striped rabbit. The elevations at the two stations were 1120 and 1455 m, and all records were taken at night (22 h33 to 03 h32), consistent with the elevational distribution range and active time of the species (Nguyen et al. 2021; Tilker et al. 2020). The distance between two stations was about 5.6 km, and the distance to the closest known records in nearby Ngoc Linh area was about 25 km. The new detected locations fall within the range map at the Map of Life (Marsh et al. 2022), but are outside the range map represented on the IUCN Red List account (Tilker et al. 2019). To the best of our knowledge, these are the first confirmed records from the Dak Long region in the Central Highlands, Vietnam.

## 4 Discussion

Dak Long is a relatively small forested area, and it is separated from the nearest known records of *N. timminsi* in Ngoc Linh by multiple roads, agricultural regions, and towns. While this result may suggest a small and localized population persists at the site, our distribution model shows that the species is likely more widely distributed in the Central Highlands, even though its populations are projected to be highly fragmented. We therefore recommend additional camera‐trapping surveys to assess Annamite striped rabbit distribution within the wider southern Annamite landscape. Based on our Maxent model, we recommend several areas, inside and outside formally recognized protected areas, for further field surveys. They include A Yun Pa Nature Reserve (Gia Lai) and nearby Dong Xuan Protection Forest (Phu Yen), Tay Hoa and Song Hinh Protection Forests (Phu Yen), the forest complex in Chu Yang Sin—Bidoup Nui Ba—Phuoc Binh National Parks (Dak Lak—Lam Dong—Ninh Thuan), and Dong Ampham National Biodiversity Conservation Area in Laos located just a few kilometers from the new records, where similar habitat is also found (Robichaud et al. 2001) (Figure 1).

Another research priority is to assess the past distribution of the species. It remains unknown if it was more widespread historically in the forest complexes in the Annamite landscape. Because of its restricted and fragmented distribution and based on its habitat preference of wet evergreen forests, we hypothesize that the rabbit's distribution range might have tracked the expansion and contraction cycles of the Annamite wet evergreen forests, which themselves followed the warmer/wetter and colder/drier cycles of the climate in Indochina. Several studies have shown in certain timeframes in the past, the wet evergreen forest in the Central Highlands regions might be much more extensive (Cannon, Morley, and Bush 2009; Sterling and Hurley 2005). Species distribution modeling can be used to project the range of the species back in time, as has been successfully predicted for other species (Cooper et al. 2016; Petersen et al. 2022).

The discovery of a new Annamite striped rabbit population based on species distribution model using Maxent emphasizes the practical application of Maxent as an integral tool for field survey planning, especially for rare and poorly‐known species. A number of studies, both in the world and in Vietnam, have yielded similar outcomes (Aizpurua et al. 2015; McCune 2016; Rhoden, Peterman, and Taylor 2017; van Schingen et al. 2016). We therefore recommend future studies to explore the possible uses of MaxEnt for field survey planning. In such cases, best practice standards, especially guidelines proposed by Blair, Le, and Xu (2022) and Sofaer et al. (2019), will be important to improve model outcomes.

The fact that we recorded the Annamite striped rabbit in an area dominated by secondary forests with moderate level of human disturbances may imply that the species may be more resilient to human activities than previously thought. Recent studies at other sites have also provided circumstantial evidences for this notion for both the Annamite striped rabbit and other endangered taxa (Cox 2024; Nguyen et al. 2023; Nguyen, Le, and Tilker 2024; Nguyen, Wilting et al. 2022). Therefore, future surveys should focus on both primary and secondary forests to have more updated and accurate information on the distribution and population of these elusive species.

Dak Long is a protection forest and is managed outside the official protected area system designated by the Vietnamese government. The protection forests are only provided with limited resources by local authorities, and they often do not receive the same level of funding, support, and research attention from the government compared to official protected areas. However, multiple surveys in recent years have revealed that despite the lack of proper investment, protection forests in Vietnam may have comparable biodiversity values, and sometimes they are even home to Critically Endangered and Endangered species (Wearn et al. 2021; Nguyen, Trinh‐Dinh et al. 2022; Le et al. 2024). Conservation and research efforts from the government and other organizations should also focus on such areas in the near future.

Our discovery of a new population of Annamite striped rabbit adds to a growing body of knowledge on its ecology and distribution, and it is welcome news for the long‐term survival of this threatened endemic species. We recommend additional surveys to better understand the population status of striped rabbits in Central Highlands. Furthermore, we believe that if properly implemented, Maxent can help identify potential areas for possible remaining and unknown populations for a suite of critically threatened and elusive species in Vietnam, such as the large‐antlered muntjac ( *Muntiacus vuquangensis* ), the Hatinh langur ( *Trachypithecus hatinhensis* ), the Owston's civet ( *Chrotogale owstoni* ), and the Vietnamese pond turtle ( *Mauremys annamensis* ).

## Author Contributions

**Anh Tuan Nguyen:** conceptualization (equal), formal analysis (lead), investigation (lead), writing – original draft (equal). **Minh Le:** conceptualization (equal), formal analysis (supporting), investigation (supporting), writing – original draft (equal).

## Conflicts of Interest

The authors declare no conflicts of interest.

## Supporting information


**Table S1.** Known localities for the Annamite striped rabbit.

## Data Availability

All data and analysis code are available at Zenodo (https://doi.org/10.5281/zenodo.14216361).
