# Supplementary material for: Modeling‐Facilitated Field Survey Discovers of a New Population of the Annamite Striped Rabbit in Kon Tum Province, Vietnam
Source: Ecol Evol. 2024 Dec 23;14(12):e70752. doi: 10.1002/ece3.70752 (PMC11664722; doi:10.1002/ece3.70752)
Supplement: Supplementary file 1 — Table S1. Known localities for the Annamite striped rabbit. [file ECE3-14-e70752-s001.docx]

**Ecology and Evolution**

**Modelling-facilitated field survey discovers of a new population of the Annamite striped rabbit in Kon Tum Province, Vietnam**

Anh Tuan Nguyen, Minh Le

**Appendix S1**

Due to high pressure from illegal hunting and trading on the Annamite striped rabbit, all coordinate data have been generalized to two decimal point, in accordance with current best practice recommendations (Chapman, 2020). Higher accuracy data is available from the authors upon reasonable request.

Table S1. Known localities for the Annamite striped rabbit

| **Scientific name** | **Latitude** | **Longitude** | **Location** (**PA**: Protected area) | **Sources** (**L**: Literature; **P**: Unpublished field data; **H**: Human observation; **C**: Camera trap record) |
| --- | --- | --- | --- | --- |
| *Nesolagus timminsi* | 18.23 | 105.06 | Ban Lak, Laos | L: (Surridge et al., 1999) |
| *Nesolagus timminsi* | 18.37 | 105.22 | Huong Son, Vietnam | L: (Averianov et al., 2000) |
| *Nesolagus timminsi* | 18.84 | 104.80 | Pu Mat PA, Vietnam | L: (Can et al., 2001) |
| *Nesolagus timminsi* | 18.56 | 105.18 | Huong Son, Vietnam | L: (Can et al., 2001) |
| *Nesolagus timminsi* | 17.45 | 106.23 | Phong Nha – Ke Bang PA, Vietnam | L: (Can et al., 2001) |
| *Nesolagus timminsi* | 16.99 | 106.68 | Dong Chau – Khe Nuoc Trong PA, Vietnam | L: (Hung et al., 2002) |
| *Nesolagus timminsi* | 17.20 | 106.40 | Quang Ninh, Vietnam | L: (Hung et al., 2002) |
| *Nesolagus timminsi* | 16.92 | 106.61 | Bac Huong Hoa PA, Quang Tri | L: (Quang Tri Forest Protection Department, 2006) |
| *Nesolagus timminsi* | 16.80 | 106.60 | Bac Huong Hoa PA, Vietnam | L: (Quang Tri Forest Protection Department, 2006) |
| *Nesolagus timminsi* | 18.29 | 105.19 | Bolikhamsai, Laos | L: (Johnson & Johnston, 2007) |
| *Nesolagus timminsi* | 17.02 | 106.47 | Le Thuy, Vietnam | L: (Anh et al., 2009) |
| *Nesolagus timminsi* | 18.41 | 105.37 | Huong Son, Vietnam | L: (Anh et al., 2009) |
| *Nesolagus timminsi* | 16.48 | 107.05 | Dak Rong PA, Vietnam | L: (Manh et al., 2009) |
| *Nesolagus timminsi* | 18.45 | 105.06 | Bolikhamsai, Laos | L: (Provincial Agriculture and Forestry Office of Bolikhamxay, 2010) |
| *Nesolagus timminsi* | 17.03 | 106.28 | Savannakhet, Laos | L: (Duckworth et al., 2010) |
| *Nesolagus timminsi* | 17.00 | 106.35 | Savannakhet, Laos | L: (Duckworth et al., 2010) |
| *Nesolagus timminsi* | 17.04 | 106.16 | Savannakhet, Laos | L: (Duckworth et al., 2010) |
| *Nesolagus timminsi* | 17.71 | 105.85 | Phong Nha – Ke Bang PA, Vietnam | L: (Bleisch et al., 2012) |
| *Nesolagus timminsi* | 16.02 | 107.71 | Nam Dong, Vietnam | L: (Schnell et al., 2012) |
| *Nesolagus timminsi* | 19.02 | 104.70 | Pu Mat PA, Vietnam | L: (Abramov et al., 2016) |
| *Nesolagus timminsi* | 16.04 | 107.34 | Xe Sap PA, Laos | L: (Tilker et al., 2020) |
| *Nesolagus timminsi* | 16.02 | 107.40 | Xe Sap PA, Laos | L: (Tilker et al., 2020) |
| *Nesolagus timminsi* | 15.97 | 107.42 | Xe Sap PA, Laos | L: (Tilker et al., 2020) |
| *Nesolagus timminsi* | 15.98 | 107.36 | Xe Sap PA, Laos | L: (Tilker et al., 2020) |
| *Nesolagus timminsi* | 12.16 | 108.43 | Bidoup – Nui Ba PA, Vietnam | L: (A. Nguyen et al., 2021) |
| *Nesolagus timminsi* | 12.14 | 108.46 | Bidoup – Nui Ba PA, Vietnam | L: (A. Nguyen et al., 2021) |
| *Nesolagus timminsi* | 12.10 | 108.36 | Bidoup – Nui Ba PA, Vietnam | L: (A. Nguyen et al., 2021) |
| *Nesolagus timminsi* | 15.24 | 107.95 | Ngoc Linh Quang Nam PA, Vietnam | L: (A. T. Nguyen et al., 2023) |
| *Nesolagus timminsi* | 15.28 | 107.72 | Ngoc Linh Kon Tum PA, Vietnam | L: (A. T. Nguyen et al., 2023) |
| *Nesolagus timminsi* | 15.22 | 107.72 | Ngoc Linh Kon Tum PA, Vietnam | L: (A. T. Nguyen et al., 2023) |
| *Nesolagus timminsi* | 15.17 | 107.92 | Ngoc Linh Kon Tum PA, Vietnam | L: (A. T. Nguyen et al., 2023) |
| *Nesolagus timminsi* | 15.47 | 107.39 | Dak Chung, Laos | L: (Rusman et al., 2023) |
| *Nesolagus timminsi* | 16.14 | 107.50 | Saola Hue PA, Vietnam | PC |
| *Nesolagus timminsi* | 15.34 | 107.73 | Phuoc Son, Vietnam | PH |
| *Nesolagus timminsi* | 15.50 | 107.60 | Song Thanh PA, Vietnam | PH |
| *Nesolagus timminsi* | 15.98 | 107.51 | Saola Quang Nam PA, Vietnam | PH |
| *Nesolagus timminsi* | 17.56 | 106.27 | Phong Nha – Ke Bang PA, Vietnam | PH |
| *Nesolagus timminsi* | 16.92 | 106.65 | Bac Huong Hoa PA, Vietnam | PH |
| *Nesolagus timminsi* | 18.74 | 104.74 | Nakai – Nam Theun PA, Laos | PH |
| *Nesolagus timminsi* | 16.96 | 106.74 | Dong Chau – Khe Nuoc Trong PA, Vietnam | PC |
| *Nesolagus timminsi* | 17.74 | 105.87 | Phong Nha – Ke Bang PA, Vietnam | PC |
| *Nesolagus timminsi* | 17.71 | 105.90 | Phong Nha – Ke Bang PA, Vietnam | PC |
| *Nesolagus timminsi* | 17.79 | 105.85 | Phong Nha – Ke Bang PA, Vietnam | PC |
| *Nesolagus timminsi* | 17.30 | 106.14 | Hin Nam No PA, Laos | PH |
| *Nesolagus timminsi* | 16.04 | 107.61 | Saola Quang Nam PA, Vietnam | PC |
| *Nesolagus timminsi* | 16.00 | 107.49 | Saola Quang Nam PA, Vietnam | PC |
| *Nesolagus timminsi* | 16.01 | 107.51 | Saola Quang Nam PA, Vietnam | PC |
| *Nesolagus timminsi* | 16.02 | 107.48 | Saola Quang Nam PA, Vietnam | PC |
| *Nesolagus timminsi* | 16.04 | 107.48 | Saola Hue PA, Vietnam | PC |
| *Nesolagus timminsi* | 16.43 | 107.24 | Phong Dien PA, Vietnam | PC |
| *Nesolagus timminsi* | 17.16 | 106.26 | Khoun Xe Nong Ma, Laos | PC |
| *Nesolagus timminsi* | 15.53 | 107.87 | Elephant PA, Quang Nam, Vietnam | PH |
| *Nesolagus timminsi* | 18.05 | 105.35 | Nakai – Nam Theun PA, Laos | PC |
| *Nesolagus timminsi* | 17.21 | 106.32 | Quang Ninh, Vietnam | PC |
| *Nesolagus timminsi* | 17.19 | 106.37 | Quang Ninh, Vietnam | PC |
| *Nesolagus timminsi* | 17.19 | 106.39 | Quang Ninh, Vietnam | PC |
| *Nesolagus timminsi* | 17.17 | 106.42 | Quang Ninh, Vietnam | PC |
| *Nesolagus timminsi* | 17.17 | 106.44 | Quang Ninh, Vietnam | PC |
| *Nesolagus timminsi* | 15.43 | 107.61 | Song Thanh PA, Vietnam | PC |
| *Nesolagus timminsi* | 15.42 | 107.64 | Song Thanh PA, Vietnam | PC |
| *Nesolagus timminsi* | 15.42 | 107.66 | Song Thanh PA, Vietnam | PC |
| *Nesolagus timminsi* | 15.44 | 107.61 | Song Thanh PA, Vietnam | PC |
| *Nesolagus timminsi* | 15.47 | 107.52 | Song Thanh PA, Vietnam | PC |
| *Nesolagus timminsi* | 15.49 | 107.50 | Song Thanh PA, Vietnam | PC |
| *Nesolagus timminsi* | 15.49 | 107.60 | Song Thanh PA, Vietnam | PC |
| *Nesolagus timminsi* | 15.51 | 107.50 | Song Thanh PA, Vietnam | PC |
| *Nesolagus timminsi* | 15.62 | 107.69 | Song Thanh PA, Vietnam | PC |
| *Nesolagus timminsi* | 16.05 | 107.46 | Saola Hue PA, Vietnam | PC |
| *Nesolagus timminsi* | 16.06 | 107.48 | Saola Hue PA, Vietnam | PC |
| *Nesolagus timminsi* | 16.05 | 107.46 | Saola Hue PA, Vietnam | PC |
| *Nesolagus timminsi* | 16.04 | 107.46 | Saola Hue PA, Vietnam | PC |
| *Nesolagus timminsi* | 15.99 | 107.62 | Saola Quang Nam PA, Vietnam | PC |
| *Nesolagus timminsi* | 15.99 | 107.62 | Saola Quang Nam PA, Vietnam | PC |
| *Nesolagus timminsi* | 16.01 | 107.56 | Saola Quang Nam PA, Vietnam | PC |
| *Nesolagus timminsi* | 16.13 | 107.52 | Saola Hue PA, Vietnam | PC |
| *Nesolagus timminsi* | 16.14 | 107.52 | Saola Hue PA, Vietnam | PC |
| *Nesolagus timminsi* | 16.14 | 107.50 | Saola Hue PA, Vietnam | PC |
| *Nesolagus timminsi* | 16.14 | 107.50 | Saola Hue PA, Vietnam | PC |
| *Nesolagus timminsi* | 16.14 | 107.50 | Saola Hue PA, Vietnam | PC |
| *Nesolagus timminsi* | 16.14 | 107.51 | Saola Hue PA, Vietnam | PC |
| *Nesolagus timminsi* | 16.13 | 107.50 | Saola Hue PA, Vietnam | PC |
| *Nesolagus timminsi* | 16.13 | 107.50 | Saola Hue PA, Vietnam | PC |
| *Nesolagus timminsi* | 16.15 | 107.51 | Saola Hue PA, Vietnam | PC |
| *Nesolagus timminsi* | 16.14 | 107.51 | Saola Hue PA, Vietnam | PC |
| *Nesolagus timminsi* | 16.14 | 107.51 | Saola Hue PA, Vietnam | PC |
| *Nesolagus timminsi* | 16.14 | 107.51 | Saola Hue PA, Vietnam | PC |
| *Nesolagus timminsi* | 16.97 | 106.68 | Dong Chau – Khe Nuoc Trong PA, Vietnam | PC |
| *Nesolagus timminsi* | 16.95 | 106.65 | Dong Chau – Khe Nuoc Trong PA, Vietnam | PC |
| *Nesolagus timminsi* | 16.97 | 106.72 | Dong Chau – Khe Nuoc Trong PA, Vietnam | PC |
| *Nesolagus timminsi* | 17.03 | 106.55 | Dong Chau – Khe Nuoc Trong PA, Vietnam | PC |
| *Nesolagus timminsi* | 17.02 | 106.55 | Dong Chau – Khe Nuoc Trong PA, Vietnam | PC |
| *Nesolagus timminsi* | 17.02 | 106.56 | Dong Chau – Khe Nuoc Trong PA, Vietnam | PC |
| *Nesolagus timminsi* | 17.02 | 106.55 | Dong Chau – Khe Nuoc Trong PA, Vietnam | PC |
| *Nesolagus timminsi* | 17.01 | 106.57 | Dong Chau – Khe Nuoc Trong PA, Vietnam | PC |
| *Nesolagus timminsi* | 17.01 | 106.56 | Dong Chau – Khe Nuoc Trong PA, Vietnam | PC |
| *Nesolagus timminsi* | 17.02 | 106.59 | Dong Chau – Khe Nuoc Trong PA, Vietnam | PC |
| *Nesolagus timminsi* | 17.01 | 106.59 | Dong Chau – Khe Nuoc Trong PA, Vietnam | PC |
| *Nesolagus timminsi* | 17.00 | 106.58 | Dong Chau – Khe Nuoc Trong PA, Vietnam | PC |
| *Nesolagus timminsi* | 17.00 | 106.57 | Dong Chau – Khe Nuoc Trong PA, Vietnam | PC |
| *Nesolagus timminsi* | 16.97 | 106.57 | Bac Huong Hoa PA, Vietnam | PC |
| *Nesolagus timminsi* | 16.99 | 106.59 | Bac Huong Hoa PA, Vietnam | PC |
| *Nesolagus timminsi* | 16.99 | 106.56 | Bac Huong Hoa PA, Vietnam | PC |
| *Nesolagus timminsi* | 16.98 | 106.57 | Bac Huong Hoa PA, Vietnam | PC |
| *Nesolagus timminsi* | 16.98 | 106.58 | Bac Huong Hoa PA, Vietnam | PC |
| *Nesolagus timminsi* | 16.97 | 106.57 | Bac Huong Hoa PA, Vietnam | PC |
| *Nesolagus timminsi* | 16.98 | 106.56 | Bac Huong Hoa PA, Vietnam | PC |
| *Nesolagus timminsi* | 16.97 | 106.58 | Bac Huong Hoa PA, Vietnam | PC |
| *Nesolagus timminsi* | 16.97 | 106.57 | Bac Huong Hoa PA, Vietnam | PC |
| *Nesolagus timminsi* | 16.97 | 106.55 | Bac Huong Hoa PA, Vietnam | PC |
| *Nesolagus timminsi* | 18.18 | 105.39 | Vu Quang PA, Vietnam | PC |
| *Nesolagus timminsi* | 18.17 | 105.40 | Vu Quang PA, Vietnam | PC |
| *Nesolagus timminsi* | 18.19 | 105.40 | Vu Quang PA, Vietnam | PC |
| *Nesolagus timminsi* | 18.18 | 105.39 | Vu Quang PA, Vietnam | PC |
| *Nesolagus timminsi* | 18.20 | 105.40 | Vu Quang PA, Vietnam | PC |
| *Nesolagus timminsi* | 18.20 | 105.37 | Vu Quang PA, Vietnam | PC |
| *Nesolagus timminsi* | 18.20 | 105.36 | Vu Quang PA, Vietnam | PC |
| *Nesolagus timminsi* | 18.21 | 105.34 | Vu Quang PA, Vietnam | PC |
| *Nesolagus timminsi* | 18.21 | 105.38 | Vu Quang PA, Vietnam | PC |
| *Nesolagus timminsi* | 16.45 | 107.20 | Phong Dien PA, Vietnam | PC |
| *Nesolagus timminsi* | 15.52 | 107.58 | Song Thanh PA, Vietnam | PC |
| *Nesolagus timminsi* | 18.55 | 105.18 | Huong Son, Vietnam | PC |
| *Nesolagus timminsi* | 16.09 | 107.49 | Saola Hue PA, Vietnam | PC |
| *Nesolagus timminsi* | 15.94 | 107.56 | Saola Quang Nam PA, Vietnam | PC |
| *Nesolagus timminsi* | 17.87 | 105.29 | Nakai – Nam Theun PA, Laos | PC |
| *Nesolagus timminsi* | 18.08 | 105.26 | Nakai – Nam Theun PA, Laos | PC |
| *Nesolagus timminsi* | 15.17 | 107.92 | Ngoc Linh Kon Tum PA, Vietnam | PH |
| *Nesolagus timminsi* | 16.05 | 107.46 | Saola Hue PA, Vietnam | PC |
| *Nesolagus timminsi* | 16.02 | 107.48 | Saola Quang Nam PA, Vietnam | PC |
| *Nesolagus timminsi* | 16.00 | 107.50 | Saola Quang Nam PA, Vietnam | PC |

**References**

Abramov, A. V, Tikhonov, A. N., & Orlov, N. L. (2016). Recent record of Annamite striped rabbit *Nesolagus timminsi* (Mammalia, Leporidae) from Vietnam. *Russian Journal of Theriology*, *15*(2), 171–174.

Anh, P. T., The, D. T., Tien, P. D., Loan, H. T., Tue, H. Van, & Son, N. T. (2009). Genetic diversity and some ecological and biological characteristics of Annamite striped rabbit (*Nesolagus timminsi*) in Vietnam. *Proceedings of the 3rd National Scientific Conference on Ecology and Biological Resources*, 1174–1179. [In Vietnamese].

Averianov, A. O., Abramov, A. V., & Tikhonov, A. N. (2000). A new species of *Nesolagus* (Lagomorpha, Leporidae) from Vietnam with osteological description. *Contributions from the Zoological Institute, Russian Academy of Sciences*, *3*, 1–22.

Bleisch, W. V., Dang, N. X., Rawson, B. M., Ha, N. M., Tuoc, D., Dat, L. T., Nghia, N. X., Tuan, N. N., & Dung, L. Van. (2012). *Biodiversity survey of mammal and gibbon and loris in and around the Phong Nha – Ke Bang National Park*. Fauna & Flora International - Vietnam Programme.

Can, D. N., Abramov, A. V., Tikhonov, A. N., & Averianov, A. O. (2001). Annamite striped rabbit *Nesolagus timminsi* in Vietnam. *Acta Theriologica*, *46*(4), 437–440. https://doi.org/10.1007/BF03192450

Chapman, A. D. (2020). *Current best practices for generalizing sensitive species occurrence data*. Copenhagen: GBIF Secretariat.

Duckworth, J. W., Hallam, C. D., Phimmachak, S., Sivongxay, N., Stuart, B. L., & Vongsa, O. (2010). *A conservation reconnaissance survey of north-east Vilabouli district, Savannakhet province, Lao PDR*. Wildlife Conservation Society.

Hung, L. M., Tien, P. D., Tordoff, A. W., & Dung, N. D. (2002). *A Rapid Field Survey of Le Thuy and Quang Ninh Districts , Quang Binh Province , Vietnam*. BirdLife International Vietnam. [In Vietnamese].

Johnson, A., & Johnston, J. (2007). *Biodiversity Monitoring And Enforcement Project In The Nam Theun 2 Watershed*. Wildlife Conservation Society: Vientiane, Lao PDR.

Manh, N. D., Dang, N. X., & Nghia, N. X. (2009). Conservation importance of mammal fauna in Dakrong Nature Reserve, Quang Tri Province. *Journal of Biology*, *31*(4), 42–50. [In Vietnamese].

Nguyen, A. T., Tilker, A., Le Khac, Q., & Le, M. (2023). New records of the Annamite striped rabbit in Ngoc Linh, Quang Nam and Kon Tum provinces, Vietnam. *Mammalia*, *87*(4), 374–378. https://doi.org/10.1515/mammalia-2023-0005

Nguyen, A., Tilker, A., Le, D., Le, H. Van, Le, S. Van, Luu, T. H., Tran, B. Van, & Wilting, A. (2021). New records and southern range extension of the Annamite striped rabbit *Nesolagus timminsi* in Vietnam. *Mammalia*, *85*(5), 417–421. https://doi.org/10.1515/mammalia-2020-0189

Provincial Agriculture and Forestry Office of Bolikhamxay. (2010). *Phou Chom Voy Provincial Protected Area management plan 2011 - 2015*. Provincial Agriculture and Forestry Office of Bolikhamxay.

Quang Tri Forest Protection Department. (2006). *Project report for establishment of Bac Huong Hoa Nature Reserve*. Quang Tri Forest Protection Department. [In Vietnamese].

Rusman, M., Sikhounmeuang, S., Phommachak, A., Pathoummavan, S., Ngonephetsy, K., Valao, M., Yoganand, K., & Tilker, A. (2023). A recent record of the Annamite striped rabbit Nesolagus timminsi in a local market in southern Lao P.D.R. *Mammalia*, 1–4. https://doi.org/10.1515/mammalia-2023-0038

Schnell, I. B., Thomsen, P. F., Wilkinson, N., Rasmussen, M., Jensen, L. R. D., Willerslev, E., Bertelsen, M. F., & Gilbert, M. T. P. (2012). Screening mammal biodiversity using DNA from leeches. *Current Biology*, *22*(8), R262–R263. https://doi.org/10.1016/j.cub.2012.02.058

Surridge, A. K., Timmins, R. J., Hewitt, G. M., & Bell, D. J. (1999). Striped rabbits in Southeast Asia. *Nature*, *400*(6746), 726–726. https://doi.org/10.1038/23393

Tilker, A., Abrams, J. Fe. H., Nguyen, A., Hörig, L., Axtner, J., Louvrier, J., Rawson, B. M., Nguyen, H. A. Q., Guegan, F., Nguyen, T. Van, Le, M., Sollmann, R., & Wilting, A. (2020). Identifying conservation priorities in a defaunated tropical biodiversity hotspot. *Diversity and Distributions*, *26*(4), 426–440. https://doi.org/10.1111/ddi.13029
